# Efficacy and safety of a new cladribine-based conditioning regimen for allogeneic hematopoietic stem cell transplantation in children with relapsed or refractory acute myeloid leukemia

**DOI:** 10.3389/fmed.2026.1747147

**Published:** 2026-03-04

**Authors:** Wen-ting Pei, Chun-lei Liu, Xiao-ling Li

**Affiliations:** 1 Department of Pediatrics, Children's Hospital Affiliated to Shandong University (Jinan Children's Hospital), Jinan, Shandong, China; 2 Department of Information Technology, Children's Hospital Affiliated to Shandong University (Jinan Children's Hospital), Jinan, Shandong, China

**Keywords:** allogeneic hematopoietic stem cell transplantation, children, cladribine, conditioning regimen, minimal residual disease, relapsed or refractory acute myeloid leukemia

## Abstract

**Objectives:**

Cladribine, a synthetic analog of deoxyadenosine, exhibits potent activity against hematological malignancies. While cladribine-containing regimens combined with allogeneic hematopoietic stem cell transplantation (allo-HSCT) have been proposed as a potential strategy to improve outcomes in relapsed or refractory (R/R) acute myeloid leukemia (AML), additional clinical evidence is needed, particularly in pediatric populations. This single-center retrospective study aimed to describe the efficacy and safety of a cladribine-based conditioning regimen for allo-HSCT in children with R/R AML.

**Materials and methods:**

Clinical data of 16 children with R/R AML who underwent allo-HSCT following a cladribine-based conditioning regimen at our hospital from October 2020 to June 2024 were analyzed retrospectively. Key outcomes included hematopoietic reconstruction, regimen-related toxicity (RRT), cumulative incidence of graft-versus-host disease (GVHD), infection profiles, overall survival (OS), disease-free survival (DFS), relapse rate, and non-relapse mortality (NRM). Flow-cytometry minimal residual disease (MRD) results before HSCT were collected and analyzed as an additional key baseline index.

**Results:**

All 16 patients attained hematopoietic reconstruction, with pre-transplant flow-cytometry MRD negative in 13 cases (81.25%) and positive (MRD ≥0.01%) in three cases (18.75%). The median time of neutrophil and platelet engraftment was 12 (10–16) days and 15 (10–25) days, respectively. The incidence of I/II grade RRT was 31.3% (oral cavity: three cases, liver: two cases), with no III/IV grade RRT observed. The cumulative incidence of acute GVHD (aGVHD) was 50.0% (grade I/II skin: five cases, grade IIIl: three cases). Among 15 evaluable patients, the cumulative incidence of chronic GVHD (cGVHD) was 26.7% (local skin: three cases, ocular keratoconjunctivitis sicca: one case). Post-transplant infections occurred in 31.3% of patients, predominantly viral pathogens: one case of BK virus-associated hemorrhagic cystitis, one case of BK virus combined with bacterial infection, and three cases of cytomegalovirus (CMV) DNAemia. The median follow-up time was 28.03 (11.67–55.34) months (follow-up cutoff: 30 June 2024). Using the Kaplan–Meier method, the 1-year OS rate was 87.5% (95% CI: 65.2%−96.4%), and the 1-year DFS rate was 87.4% (95% CI: 64.9%−96.3%). The relapse rate and NRM were both 6.3% (95% CI: 0.8%−29.1%); the NRM case was confirmed as bronchiolitis obliterans syndrome (BOS) induced by pulmonary cGVHD.

**Conclusion:**

In this small single-center retrospective series, the cladribine-based conditioning regimen was associated with favorable hematopoietic reconstruction, mild RRT, and promising survival outcomes in children with R/R AML, even in partial patients with pre-transplant MRD positivity. However, due to the limited sample size, single-arm design, and lack of a control group, conclusions regarding superiority (e.g., improved OS or reduced relapse) cannot be drawn. Larger prospective multi-center studies are required to validate these preliminary findings.

## 1 Introduction

Acute myeloid leukemia (AML), characterized by abnormal hematopoietic stem cell proliferation and differentiation suppression, accounts for approximately 20% of childhood leukemia cases, and recent clinical studies have reported an overall 5-year survival (OS) rate exceeding 70% in pediatric AML (1). In contrast, the prognosis for relapsed/refractory AML (R/R-AML) remains poor, with only 20%−30% event-free survival (EFS) (2). Allogeneic hematopoietic stem cell transplantation (allo-HSCT) emerges as a promising therapeutic avenue, aiming at a curative approach (3). However, post-transplant relapse remains the most important factor affecting patient survival (4). The intensive conditioning regimens have a stronger anti-leukemia effect, which can eliminate more residual leukemia cells, thus reducing the relapse rate after transplantation. Currently, the widely adopted intensive conditioning regimen is the busulfan (Bu) plus cyclophosphamide (Cy)-based approach, with alternative regimens including Cy plus total body irradiation, fludarabine plus Bu, cytarabine-based, and mitoxantrone-based regimens (5).

Cladribine, a synthetic analog of deoxyadenosine utilized for hematological malignancies, can increase the sensitivity of residual abnormal cells to chemotherapy drugs, consume DNA methyltransferase donors to induce apoptosis, and induce a significant lymphocyte-depleting effect. Furthermore, cladribine induces apoptosis in non-dividing cells, contributing to a stronger suppressive effect on malignant clones (6, 7). Its application extends to a rescue therapy for R/R-AML. In search of an effective intensive conditioning regimen for R/R-AML, we attempted to add cladribine to the classic conditioning regimen, aiming to enable AML patients to achieve maximal complete remission (CR) and minimal residual disease (MRD) negativity before allo-HSCT to achieve greater transplantation benefit. Recently, cladribine for allo-HSCT in AML has shown certain prospects and has been mainly used in adult populations. Only a small number of studies have suggested that cladribine plus BuCy may serve as an intensive conditioning regimen for children with R/R AML, and the sample size of these studies is relatively small. This retrospective analysis of 16 children with R/R-AML aims to provide preliminary clinical data on the efficacy and safety of a cladribine-based conditioning regimen, acknowledging the study's limitations.

## 2 Materials and methods

### 2.1 Study design and patient selection

In this retrospective study, 16 patients with R/R AML between October 2020 and June 2024 were enrolled. Inclusion criteria comprised: (1) diagnosis of R/R AML following the *Guidelines for Diagnosis and Treatment of Acute Myelogenous Leukemia (Relapse/Refractory) in China (2017)* (8); (2) age ≤ 18 years; (3) underwent allo-HSCT with a cladribine-based conditioning regimen; and (4) complete clinical and laboratory data, including pre-transplant flow-cytometry MRD results. Exclusion criteria included: (1) concurrent solid tumor or other malignant hematologic disease and (2) incomplete clinical records. Clinical data collected included baseline characteristics, transplantation details, hematopoietic reconstruction, complication prophylaxis, and post-transplant outcomes.

### 2.2 Cladribine-based conditioning regimen

The cladribine-based conditioning regimen was administered as follows, with dosing scheduled by days −11 to −1 (pre-transplant): cladribine 5 mg/(m^2^·d) on days −11 to −9, cytarabine 2 g/(m^2^·d) on days −11 to −9, Bu 3.2 mg/(kg·d) on days −8 to −6, etoposide 300 mg/(m^2^·d) on days −5, rabbit antithymocyte globulin (ATG) 2.5 mg/(m^2^·d) (HLA-matched donors)/(7.5–8.5) mg/(m^2^·d) (HLA-partially matched donors) on days −5 to −2, and Cy 40 mg/(kg·d) on days −4 to −2. For patients with central nervous system (CNS) leukemia, lomustine (CCNU) 250 mg/(m^2^·d) was added on days −11. The regimen-related toxicity (RRT) was assessed according to Bearman criteria, which included cardiac, pulmonary, hepatic, renal, bladder, oral mucosa, gastrointestinal, and CNS.

### 2.3 Graft versus host disease (GVHD) prophylaxis

GVHD prophylaxis regimens were stratified by HLA matching status, as detailed in Table 1. GVHD grading and diagnosis were performed in accordance with the 2014 NIH Consensus Development Project criteria for cGVHD and the modified Glucksberg criteria for aGVHD—diagnosis was based on clinical symptoms, physical examination findings, and organ-specific laboratory/imaging evidence, with no strict 100-day post-transplant cutoff for distinguishing aGVHD and cGVHD (9). aGVHD was defined by acute-phase inflammatory manifestations in target organs (skin, gastrointestinal tract, and liver) with no chronic fibrotic changes; cGVHD was defined by chronic fibrotic or sclerotic manifestations in target organs, which may occur *de novo* or follow aGVHD. The severity of aGVHD and cGVHD was graded according to the above consensus criteria (grade I–IV for aGVHD; limited or extensive for cGVHD).

**Table 1:** GVHD prophylaxis regimens stratified by HLA matching status.

**HLA matching status**	**Prophylaxis regimen**	**Dosing details**
Matched	CsA + short-course MTX	2.5 mg/(kg·d) on day 7 pre-transplantation and 3–5 mg/(kg·d) twice daily after hematopoietic reconstruction; MTX: 15 mg/(m^2^) on d+1, 10 mg/(m^2^) on d+3, +6, +11
Partially matched	CsA + MMF + anti-CD25 monoclonal antibody	CsA: same as above; MMF: 300 mg/(m^2^) every 12 h on day 7; anti-CD25 mAb: 20 mg on d+1 and d+4

MTX, methotrexate; MMF, mycophenolate mofetil; mAb, monoclonal antibody.

### 2.4 Infection prophylaxis

The specific regimens were as follows: pre-transplantation, both donors and recipients are tested for antibodies to cytomegalovirus (CMV), EB virus (EBV), and herpes simplex virus (HSV) pre-transplantation; ganciclovir for preventing viral infections; and oral cephalosporin antibiotics and gentamicin for the elimination of intestinal infection. Post-transplantation, posaconazole for preventing fungal infections and acyclovir for preventing viral infections. After hematopoietic reconstruction, trimethoprim-sulfamethoxazole is used to prevent *Pneumocystis jirovecii* infection.

Infections were defined as clinical infections requiring intravenous antimicrobial treatment or laboratory-confirmed pathogenic infection (excluding asymptomatic viral reactivation). CMV infection was categorized as CMV DNAemia (positive CMV DNA without clinical symptoms) or CMV disease (DNAemia plus organ-specific symptoms).

### 2.5 Transplantation efficacy

The first of three consecutive days in which the absolute neutrophil count was >0.5 × 10^9^/L was defined as neutrophil engraftment. The time of platelet reconstitution was defined as the first of seven consecutive days in which platelets were >20 × 10^9^/L without transfusion support, which was defined as platelet engraftment (10). Pre-transplant MRD was detected by multicolor flow cytometry (MFC), with a positivity threshold of ≥0.01%. Bone marrow (BM) morphology, minimal residual disease (MRD), and short tandem repeat polymorphism (STR) were performed on day 30 post-transplantation to detect engraftment and remission status. The above detection was monitored at months 1, 2, 3, 4, 5, 6, 9, and 12 post-transplantation, every 3 months in the second year, and every 6 months thereafter for 5 years. CR was defined as morphologic remission (<5% BM blasts). Relapse was defined as morphologic evidence of leukemia in the bone marrow or any extramedullary site, or MRD positivity (≥0.01%) with clinical progression.

### 2.6 Follow-up

The endpoint for follow-up was 30 June 2024. Overall survival (OS) was defined as the time from transplantation to death due to any cause or censored at last follow-up. Disease-free survival (DFS) was defined from allo-HSCT to relapse, death from any cause, or last follow-up (censored). Non-relapse mortality (NRM) was defined as death after transplantation due to treatment-related side effects or complications, rather than the recurrence of the primary disease.

### 2.7 Ethical statement

This study was conducted in accordance with good clinical practice guidelines and the Declaration of Helsinki. The study protocol was approved by the local ethics committee, which waived the requirement for obtaining informed consent due to the retrospective nature of the study.

### 2.8 Statistical analysis

Quantitative data with normal distribution were expressed as mean ± standard deviation (SD); non-normal data as median (range). Hematopoietic reconstruction time, RRT incidence, and infection profiles were described using descriptive statistics. OS and DFS were estimated by the Kaplan–Meier method with 95% confidence intervals (95% CI). Cumulative incidence of GVHD was calculated using competing risk analysis (competing event: death without GVHD). A two-sided *P*-value of <0.05 was considered statistically significant. Analyses were performed using IBM SPSS Statistics 26.0 and R (programming language for statistical computing) 4.2.0.

## 3 Results

### 3.1 Patient characteristics and hematopoietic reconstruction

The median age of the 16 patients was 8.3 (2–15) years, with 10 males (62.5%) and six females (37.5%). Six (37.5%) patients were diagnosed with relapsed AML, and 10 (62.5%) with refractory AML. All patients achieved CR pre-transplant; flow-cytometry MRD detection showed 13 cases (81.25%) with MRD negativity (<0.01%) and 3 cases (18.75%) with MRD positivity (0.03%−0.12%). Fourteen (87.5%) patients had negative fusion genes; among four patients with RUNX1-RUNX1T1 positivity, two had transcript levels >0.05% pre-transplant (case 6: 0.32%; case 13: 0.76%), and both were MRD positive by MFC.

Twelve (75.0%) patients underwent peripheral blood stem cell (PBSC) transplantation, and 4 (25.0%) received bone marrow stem cells plus PBSC transplantation. HLA was partially matched in 14 (87.5%) patients and matched in the remaining 2 (12.5%) patients. The median mononuclear cell (MNC) and CD34+ cells were 9.67 (8.21–11.53) × 10^8^/kg and 7.12 (5.74–10.32) × 10^6^/kg, respectively.

After allo-HSCT, all patients achieved hematopoietic reconstruction, with a median time of 12 (10–16) days for neutrophil reconstitution and 15 (10–25) days for platelet reconstitution. Morphology and MRD of the bone marrow indicated the CR state at 30 days post-transplantation, as detailed in Tables 2, 3.

**Table 2:** Transplant characteristics and hematopoietic reconstitution in 16 R/R AML patients.

**No**.	**Sex**	**Age (year)**	**Cytogenetics**	**Molecular biology**	**MRD**	**HLA matching**	**Source of stem cells**	**MNC dose (× 10^8/^L)**	**CD34+dose (× 10^6/^L)**	**Neutrophils engraftment (d)**	**Platelet engraftment (d)**
1	Female	15	46,XY	KRAS, WT1mutation	<0.01%	6/12	PB	11.53	10.32	10	10
2	Female	9	46,XX, t(8;10;21) (q22;q26;q22) [16]/46,XX[4]	RUNX1-RUNX1T1 (+), CEBPA mutation	<0.01%	10/10	PB	9.21	7.09	14	16
3	Male	4	46,XY, t(5;11) (q31;p15)[17]/ 46,XY[3]	MLL-AF9 (+)	<0.01%	8/10	PB	8.34	6.13	13	17
4	Male	2	45,X,-Y, t(8;21) (q22;q22)	RUNX1-RUNX1T1 (+), PTPN11 mutation	<0.01%	6/12	PB	8.21	6.74	15	21
5	Male	9	46,XY, del(11)(q23)	MLL-ENL (+)	<0.01%	7/12	BM+PB	10.13	7.16	11	15
6	Male	11	46,XY, t(8;21) (q22;q22)	RUNX1-RUNX1T1 (+), C-KIT mutation	0.07%	10/10	PB	11.44	7.28	13	14
7	Female	5	46,XX	FLT3, PTPN11, KRAS mutation	<0.01%	8/10	PB	9.13	6.01	16	25
8	Male	6	46,XY, inv(16)(p13.1q22)	WT1, CBFB-MYH11 mutation	<0.01%	6/12	BM+PB	9.37	7.62	14	15
9	Male	8	46,XY	MLL-AF9 (+), BRAF mutation	0.03%	6/12	PB	9.97	6.45	11	12
10	Male	9	46,XY,t(7;11) (q36;q22), t(8:21)(q22;q22)	MLL-PTD (+), WT1 mutation	<0.01%	7/12	PB	9.73	6.92	12	16
11	Female	5	45,X,-X, der(11) t(11;18)(q14;q23), +18	KRAS, TET-2 mutation	<0.01%	6/12	PB	8.91	7.20	11	14
12	Male	9	46,XY, t(1;11)(p32;q23) [13]/47, idem,+9[7]	MLL-AF11 (+), WT1, EV11 mutation	<0.01%	7/12	PB	9.30	7.55	12	15
13	Female	7	46,XX,t (8;21)(q22;q22)	RUNX1-RUNX1T1 (+), TET2 mutation	0.12%	6/12	BM+PB	10.11	7.34	12	15
14	Female	10	46,XX	PTPN11, WT1 mutation	<0.01%	6/12	PB	9.59	6.16	12	17
15	Male	12	46,XY	WT1, CEBPA, FLT3-ITD mutation	<0.01%	7/12	BM+PB	10.28	7.32	13	13
16	Male	10	46,XY	KRAS, WT1 mutation	<0.01%	6/12	PB	9.47	6.63	11	14

R/R, relapsed or refractory; AML, acute myeloid leukemia; PB, peripheral blood; BM, bone marrow; HLA, human leukocyte antigen; MNC, monocyte cell.

**Table 3:** Clinical characteristics of 16 R/R AML patients.

**Items**	**R/R AML patients**
**Sex**, ***n*** **(%)**	
Male	10 (62.5)
Female	6 (37.5)
Age (years), median (range)	8.3 (2–15)
**AML type**, ***n*** **(%)**	
Relapsed	6 (37.5)
Refractory	10 (62.5)
**HLA matching**, ***n*** **(%)**	
Matched	2 (12.5%)
Partially matched	14 (87.5%)
MNC dose (× 10^8^/L), median (range)	9.67 (8.21–11.53)
CD34+ dose (× 10^6^/L), median (range)	7.12 (5.74–10.32)

R/R, relapsed or refractory; AML, acute myeloid leukemia; HLA, human leukocyte antigen; MNCs, monocyte cells.

### 3.2 RRT and GVHD

Table 4 provides a detailed RRT occurrence. Five (31.3%) patients experienced grade I/II RRT, including three cases in the oral cavity and two cases in the liver. It mostly occurred within 2 weeks of the conditioning regimen, and could be effectively improved after symptomatic treatment. No grade III/IV RRT and no adverse reactions resulting in death related to the conditioning regimen in the patients were reported.

**Table 4:** Regimen-related toxicity of patients with R/R AML according to Bearman's criteria.

**Organ and system**	**Grade I/II**	**Grade III/IV**
Oral cavity	3	0
Heart	0	0
Liver	2	0
Lung	0	0
Kidney	0	0
Gastrointestinal tract	0	0
Bladder	0	0
Central nervous system	0	0

R/R, relapsed or refractory; AML, acute myeloid leukemia.

The cumulative incidence of aGVHD was 50.0% (8/16 patients), with severity grading as shown in Table 5; no grade IV aGVHD was observed. Among the 15 patients evaluable for cGVHD (1 lost to follow-up before cGVHD assessment), the cumulative incidence was 26.7% (4/15 patients); the cGVHD manifestations included three cases of limited local skin cGVHD and one case of ocular keratoconjunctivitis sicca (limited cGVHD). No hepatic vein occlusion disease was reported in any patients.

**Table 5:** Severity grading of aGVHD in 16 pediatric R/R AML patients.

**aGVHD grade**	**Number of cases (*n*)**	**Proportion (%)**	**Involved organ**
I/II	5	31.25	Skin only
III	3	18.75	Gastrointestinal tract
IV	0	0	–
Total aGVHD	8	50	

R/R, relapsed or refractory; AML, acute myeloid leukemia.

### 3.3 Infection prophylaxis

A total of five (31.3%) patients experienced infections, with the virus as the main pathogen. One case of BK virus and one case of BK combined with bacteria were included, all of whom developed grade II hemorrhagic cystitis. During follow-up, three cases of CMV DNAemia (no clinical symptoms, resolved with ganciclovir). No EBV viremia, sepsis, or fatal infections were observed.

### 3.4 Survival

The median follow-up time was 28.03 (11.67–55.34) months. Up to the last follow-up, 14 patients (87.5%) survived, and two patients (12.5%) died. One death was due to disease relapse (relapse rate: 6.3%, 95% CI: 0.8%−29.1%), and the other was confirmed as bronchiolitis obliterans syndrome (BOS) induced by pulmonary extensive cGVHD (NRM: 6.3%, 95% CI: 0.8%−29.1%); this pulmonary cGVHD case had no prior aGVHD and presented *de novo* fibrotic changes in the lungs at 6-month post-transplant.

**Figure 1:**
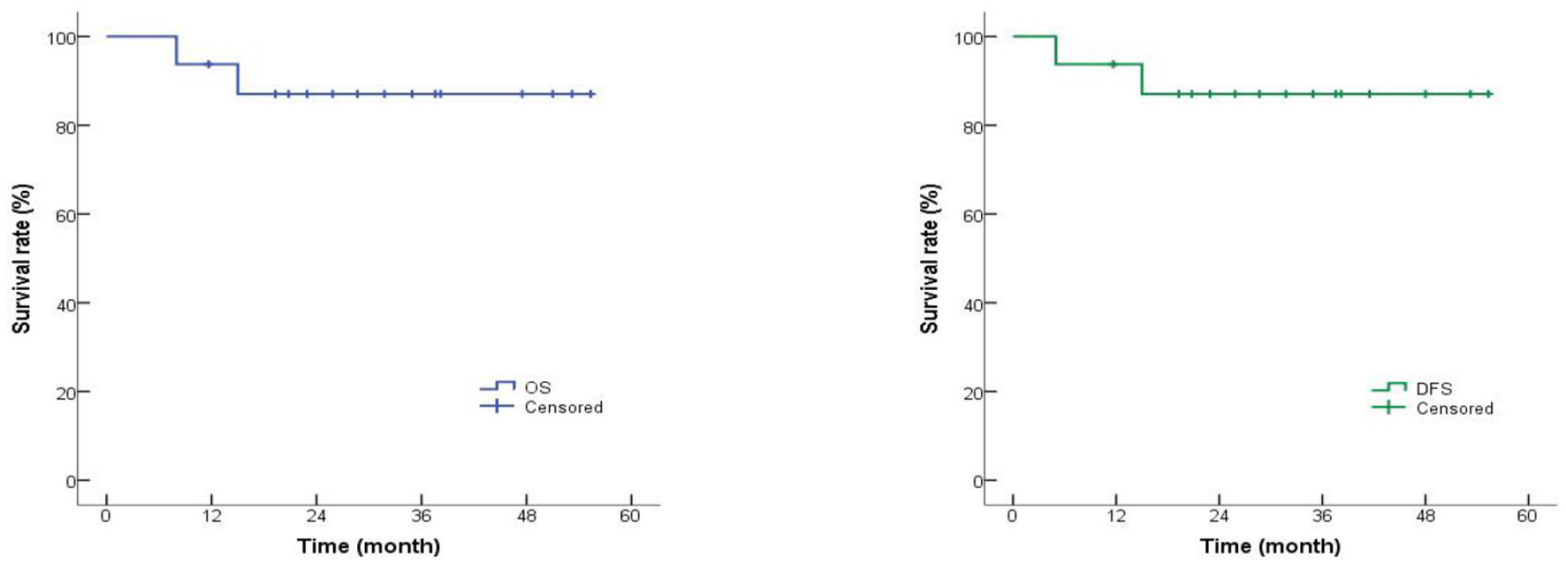
Overall survival (OS) and disease-free survival (DFS) rates of R/R AML patients. The 1-year OS rate was 87.5% (95% CI: 65.2%−96.4%), and the 1-year DFS rate was 87.4% (95% CI: 64.9%−96.3%).

Using the Kaplan–Meier method, the 1-year OS rate was 87.5% (95% CI: 65.2%−96.4%), and the 1-year DFS rate was 87.4% (95% CI: 64.9%−96.3%; Figure 1). The two patients with pre-transplant RUNX1-RUNX1T1 positivity (cases 6 and 13) had negative fusion gene transcripts and MRD negativity 1-month post-transplant, with no relapse at the last follow-up.

## 4 Discussion

### 4.1 Conditioning regimen of allo-HSCT on R/R AML

R/R AML is clinically challenging to treat due to its short survival, multiple complications, and difficulty sustaining complete remission. Although novel anti-AML drugs have improved re-induction remission rates in some patients, the long-term efficacy remains unsatisfactory. Allo-HSCT is currently the only curative treatment for R/R AML, especially for patients with primary drug resistance or early recurrence, as it can eradicate leukemia cells via pre-transplant conditioning and post-transplant graft-versus-leukemia (GVL) effect. Myeloablative conditioning (MAC) exerts a stronger GVL effect but is associated with higher GVHD and transplant-related mortality; reduced-intensity conditioning (RIC) reduces transplant-related mortality but fails to lower GVHD and leukemia relapse rates (11). The coexistence of GVHD and GVL is a major clinical challenge, and balancing pre-transplant toxicity and GVL effect is the core of optimizing conditioning regimens. Scott et al. (12) conducted a long-term follow-up of the BMT CTN 0901 trial and found that RIC for AML/MDS had lower transplant-related mortality but inferior DFS compared with MAC, suggesting that MAC should be the first choice for AML/MDS patients with good performance status. This finding supports our use of a cladribine-based intensified MAC regimen for pediatric R/R AML.

### 4.2 Cladribine-based conditioning regimen: direct comparison with published studies

#### 4.2.1 Hematopoietic reconstruction

Our study showed that all 16 pediatric patients achieved hematopoietic reconstruction, with median neutrophil and platelet engraftment times of 12 and 15 days, respectively. This result is comparable and slightly superior to published adult studies: Xiao et al. (13) reported median neutrophil and platelet engraftment times of 14 and 13 d in 23 adult R/R AML patients with cladribine + BuCy conditioning; Shi et al. (14) reported median neutrophil and platelet engraftment times of 13 d and 28 d in adult AML patients with a cladribine-containing regimen for auto-HSCT. The slightly faster neutrophil engraftment in our pediatric cohort may be related to the immature hematopoietic microenvironment in children, which has a stronger regenerative capacity, and the optimal stem cell infusion dose in our study.

#### 4.2.2 RRT and GVHD

The incidence of I/II grade RRT in our study was 31.3%, with no III/IV grade RRT. This is significantly lower than the 69.6% I/II grade CRRT incidence reported in adult R/R AML patients with cladribine + BuCy conditioning (13). In terms of organ-specific toxicity, our hepatic toxicity incidence (12.5%) was similar to the 27.3% grade I/II liver CRRT in adult studies, and no gastrointestinal RRT was observed (vs. 100% gastrointestinal CRRT in adult studies) (14), which may be due to the optimized dosing schedule of cladribine and cytarabine in our regimen, reducing cumulative gastrointestinal mucosal damage.

For GVHD, the cumulative incidence of aGVHD in our study was 50.0% (grade III: 18.75%, no grade IV), and cGVHD was 26.7%—these rates are consistent with the range of adult studies (aGVHD: 16.7%−50.0%; cGVHD: 13.0%−44.4%) (14, 15). Notably, our study used the 2014 NIH criteria for GVHD diagnosis (excluding the 100-day cutoff), and the only cGVHD-related death was confirmed as BOS, a severe manifestation of pulmonary cGVHD that is rare in pediatric cohorts. This highlights the need for long-term monitoring of pulmonary function in pediatric patients after allo-HSCT. One case of ocular cGVHD in our study was diagnosed as keratoconjunctivitis sicca, the most common ocular manifestation of cGVHD, which is consistent with adult clinical findings (14, 15).

#### 4.2.3 Infections

Post-transplant infection incidence in our study was 31.3%, with viral pathogens as the main cause. This rate is lower than the reported infection incidence in adult cladribine-based conditioning studies (≥50%) (13, 15). No fatal infections or sepsis were observed, which may be attributed to strict pre-transplant donor/recipient viral screening, standardized anti-infective prophylaxis, and milder RRT in our pediatric cohort (reducing the duration of neutropenia). The three cases of asymptomatic CMV DNAemia were all resolved with ganciclovir, indicating that the anti-viral prophylaxis regimen is effective for pediatric patients.

#### 4.2.4 Survival outcomes

Our pediatric cohort achieved a 1-year OS rate of 87.5%, 1-year DFS rate of 87.4%, and NRM of 6.3%. These results are markedly superior to adult R/R AML studies with cladribine-based conditioning: Xiao et al. (13) reported 1-year OS/2-year OS of 74.7%/64.0% and 1-year/2-year EFS of 66.3%/53.1%; Sun et al. (15) reported a 2-year OS of 61.4% and NRM of 9.1%; Tong et al. (16) reported a 1-year OS of 85.6% and 1-year RFS of 73.8%. The superior survival in our study may be due to two factors: (1) cladribine has higher transport efficiency in pediatric cells via human equilibrative and concentrative nucleoside transporters, leading to a stronger anti-leukemia effect (17). (2) All our patients achieved morphologic CR pre-transplant, with 81.25% MRD negative, and the three MRD-positive patients all achieved MRD negativity post-transplant, reducing the relapse risk. In addition, the low NRM (6.3%) in our study is related to mild RRT and low severe infection incidence, further improving survival outcomes.

### 4.3 RUNX1-RUNX1T1 fusion gene positive

Translocation t (8;21) (q22; q22) (RUNX1-RUNX1T1) accounts for 10%−15% of pediatric AML and is a favorable cytogenetic subtype in *de novo* AML (18). However, RUNX1-RUNX1T1-positive patients with additional adverse prognostic factors (e.g., c-kit and TET2 mutations) have a poor prognosis, and allo-HSCT can significantly improve their survival (19). Tarantino et al. (20) reported that additional cytogenetic abnormalities and RIC are associated with increased relapse risk in RUNX1-RUNX1T1-positive AML patients undergoing allo-HSCT. In our study, two RUNX1-RUNX1T1-positive patients with additional mutations (c-KIT/TET2) and pre-transplant MRD positivity achieved negative fusion gene transcripts and MRD negativity 1-month post-transplant with the cladribine-based MAC regimen, with no relapse at the last follow-up. This result is better than the reported relapse rate of 20%−30% in high-risk t (8;21) pediatric AML (19), suggesting that the cladribine-based intensified MAC regimen can effectively eliminate residual leukemia cells in high-risk RUNX1-RUNX1T1-positive pediatric R/R AML. However, the small number of such patients in our study (*n* = 4) means that more clinical data are needed to confirm this conclusion.

### 4.4 Study limitations

This study has several critical limitations: (1) single-center retrospective design with a small sample size (*n* = 16), leading to limited statistical power and potential selection bias; (2) lack of a control group (e.g., historical controls treated with standard BuCy), precluding definitive conclusions about efficacy superiority; (3) no multivariate analysis (removed due to inappropriate application with *n* = 16 and two events); and (4) limited generalizability due to single-arm design and differences in patient populations compared to literature. Future prospective multi-center randomized controlled trials are needed to validate these findings.

## 5 Conclusion

In this small single-center retrospective study, the cladribine-based conditioning regimen was associated with successful hematopoietic reconstruction, mild RRT, manageable infections, and promising survival outcomes in children with R/R AML, even in partial patients with pre-transplant MRD positivity and high-risk genetic subtypes (e.g., RUNX1-RUNX1T1 with additional mutations). The regimen also effectively induced MRD negativity in post-transplant bone marrow and reduced relapse risk in pediatric patients. However, due to the study's limitations (small sample size, single-center, no control group), claims of improved OS or reduced relapse rates compared with the standard BuCy regimen are unwarranted. The regimen warrants further investigation in larger prospective controlled trials to confirm its efficacy and safety in pediatric R/R AML, and long-term monitoring of pulmonary cGVHD (e.g., BOS) is recommended for pediatric patients after allo-HSCT.

## Data Availability

The original contributions presented in the study are included in the article/supplementary material, further inquiries can be directed to the corresponding author.
